# Phenotypic and Genotypic Shifts in Hepatitis B Virus in Treatment-Naive Patients, Taiwan, 2008–2012

**DOI:** 10.3201/eid2305.161894

**Published:** 2017-05

**Authors:** Chau-Ting Yeh, Kung-Hao Liang, Ming-Ling Chang, Chao-Wei Hsu, Yi-Cheng Chen, Chih-Lang Lin, Wey-Ran Lin, Ming-Wei Lai

**Affiliations:** Chang Gung Memorial Hospital, Taoyuan, Taiwan

**Keywords:** phenotype, genotype, shifts, hepatitis B virus, treatment-naive patients, Taiwan, viruses

## Abstract

We examined the characteristic changes of hepatitis B virus (HBV) in antiviral drug treatment–naive patients referred for pretreatment evaluation in Taiwan during 2008–2012. Over time, we observed substantial decreases in the prevalence of HBV e antigen (HBeAg) and increasing prevalence of the precore G1899A mutation and HBV-DNA levels in HBeAg-positive patients.

Hepatitis B virus (HBV) replication is dependent on the activity of its reverse transcriptase, an error-prone enzyme, which results in the accumulation of genomic mutations. In the natural course of chronic hepatitis B, the prevalence of some mutations (e.g., precore stop codon mutations, basal core promoter mutations, deletions in the pre-S gene region) gradually increases with the progression of disease through the different clinical stages (*1*,*2*). This increase is largely caused by longer duration of chronic HBV infection, during which HBV has to adapt to environmental changes for better survival. Because of the availability of antiviral drug therapies, HBV replication can now be completely suppressed in most patients (*3*,*4*). However, drug resistance and suboptimal or no responses to antiviral drugs can occur (*5*), and there is a lag of few months between the start of treatment and complete virologic suppression. In addition, treatment noncompliance and intermittent treatment almost always result in virologic, or even clinical, relapses. In such cases, antiviral drug therapy imposes an iatrogenic selection pressure on HBV, and the selected viruses carrying mutations can cause infections.

HBV has been infecting humans for a long time; thus, it is possible that genotypic or phenotypic changes have occurred over time, especially since the introduction of HBV vaccine and antiviral drugs. We examined serologic changes and genotypic alterations of HBV in treatment-naive patients during 2008–2012 in Taiwan, where a universal vaccination program was launched in 1986.

## The Study

During January 2008–December 2012, we reviewed the clinical and virologic data for 1,224 treatment-naive patients with chronic hepatitis B who were referred to our clinic at Chang Gung Memorial Hospital, Taoyuan, Taiwan, from all parts of Taiwan for pretreatment evaluation. We obtained patients’ age and sex and assessed platelet count, cirrhosis status, HBV e antigen (HBeAg) and HB e antibody (anti-HBe) status, HBV genotype, HBV DNA level, basal core promoter mutations, and precore stop codon mutations. We conducted the study using previously described methods (*6*,*7*). The study was approved by the Institutional Review Board of Chang Gung Memorial Hospital.

Univariate regression analysis indicated a significant increase in patient age (p = 0.001) and a significant decrease in the number of HBeAg-positive patients (p<0.001) over the 5-year year period. Three factors were significantly associated with HBeAg status: an increased prevalence of anti-HBe (p = 0.004) and increased prevalence of precore G1896A (p = 0.003) and G1899A (p = 0.019) mutations. However, no significant changes occurred in the prevalence of 9 of 10 basal core promoter mutations (Technical Appendix Table 1). We noted a mild, but significant, decrease in the prevalence of G1730C mutations (p = 0.034).

Multivariate analysis showed that patient age and changes in the prevalence of HBeAg were independent of each other (adjusted p = 0.025 and 0.021, respectively). However, multivariate analysis that included G1730C, HBeAg, and age showed that the changing prevalence of G1730C was not independent of HBeAg and age (adjusted p = 0.222, 0.049, and 0.027, respectively).

These data indicate that the decreasing prevalence of HBeAg was not due to the increasing age of treatment-naive patients but due to an authentic phenotypic change of HBV over the years. It was unclear why patient age increased over the 5-year period; one possibility is the gradual acceptance of antiviral drug therapy by older patients, who may have been worried about possible side effects of new drugs. The cause of the decreasing prevalence of HBeAg over time was possibly due to the fact that a higher proportion of HBeAg-positive patients were treated in the early era of antiviral drug use and more HBeAg-negative patients received treatment at a later time. Alternatively, because HBV infection has occurred in and expanded among the human population over the past decades, the serologic alterations may be attributed to a changing trend in the mode of transmission on a population scale; for example, in Taiwan, horizontal transmission has increased because of injection drug abuse and sexual transmission, and vertical transmission has decreased because of the neonatal vaccination program.

We subsequently separated patients into HBeAg-positive (n = 398) and HBeAg-negative (n = 826) groups. In the HBeAg-positive group, we saw a significant increase over time in the prevalence of precore G1899A mutations (p = 0.039) and level of HBV DNA (p = 0.013); however, these 2 factors were independent of each other (adjusted p = 0.009 and 0.003, respectively). Of note, we found no change in age over time in this subgroup (p = 0.281) (online Technical Appendix Table 2). Furthermore, because these patients were all HBeAg-positive, we could not explain the data by a differential proportion of HBeAg-positive patients being treated in the early and later periods of the antiviral drug era. Instead, other factors (e.g., changes in transmission routes, altered predominant risks of exposure, changes of HBV prevalence in different subpopulations) could be responsible. Alternatively, dependent on the scale of antiviral drug treatment received in this population, the therapeutic methods might also partly contribute to selection of mutation G1899A and HBV with higher replication efficiency.

In the HBeAg-negative treatment-naive patients (n = 826), we found a borderline increase in patient age (p = 0.046) and a borderline decreased prevalence of male patients (p = 0.044) over time. In addition, we noted a significant decrease in the prevalence of mutation A1752G over the 5-year period (p = 0.022). Multivariate analysis showed that these 3 changes were not independent (adjusted p = 0.062 for age, 0.067 for male sex, and 0.201 for mutation A1752G; data not shown).

## Conclusions

Our findings showed a shift in the phenotypic and genotypic characteristics of HBV in treatment-naive patients in Taiwan, an area where chronic hepatitis B is endemic, after the widespread use of antiviral drugs. In Taiwan, because of a limited budget for national health insurance and a high prevalence of chronic hepatitis B, insurance coverage for hepatitis B treatment is not lifelong. Under the insurance plan, patients are provided with continuous nucleotide and nucleoside antiviral treatment for 3 years, after which the drugs are withdrawn to observe for durability of clinical remission. About half of patients have clinical relapses, and almost all have virologic relapses (*8*). Only patients with clinical relapses are retreated. After 3 years of retreatment, the antiviral drugs are withdrawn again to observe for durability; subsequent retreatments (for 3 years) are given only to patients with clinical relapses. The procedure is repeated until no clinical relapse has occurred. It is conceivable that the characteristics of HBV in these antiviral drug–treated, hepatitis B–relapsed patients have been selected by antiviral drugs. Antiviral drug–selected HBVs could potentially spread to treatment-naive patients and cause new infection or superinfection. However, the contribution of this therapeutic factor to the phenotypic and genotypic alterations of HBVs is unclear. Other environmental and social factors, such as altered transmission routes, changes of exposure risks, variations of HBV prevalence in subpopulations over time, and effects of neonatal vaccination, could lead to the changes in HBV characteristics.

Over the 5 years of this study, we found an increasing prevalence of G1899A mutations and an increasing concentration of serum HBV DNA in treatment-naive, HBeAg-positive patients. The mean HBV DNA concentrations increased from 7.7 to 8.3 log_10_/mL and then stabilized without further increases during the last 3 years of the study. On the other hand, prevalence of G1899A mutations increased from 2.5% to 17.4%. The clinical significance of this mutation in HBeAg-positive patients is unclear and requires further study. In conclusion, this study revealed that, in Taiwan, HBV characteristics have been changing in the era after introduction of antiviral drug treatment and HBV vaccination.

Technical Appendix Demographic, clinical, and virologic characteristics of treatment-naive patients with chronic hepatitis B, and clinical and virologic characteristics of 398 treatment-naive, hepatitis B virus e antigen–positive patients Taiwan, 2008–2012.
