# Supplementary material for: Phenotypic and Genotypic Shifts in Hepatitis B Virus in Treatment-Naive Patients, Taiwan, 2008–2012
Source: Emerg Infect Dis. 2017 May;23(5):820–1. doi: 10.3201/eid2305.161894 (PMC5403058; doi:10.3201/eid2305.161894)
Supplement: Technical Appendix — Demographic, clinical, and virologic characteristics of treatment-naive patients with chronic hepatitis B, and clinical and virologic characteristics of 398 treatment-naive, hepatitis B virus e antigen–positive patients Taiwan, 2008–2012. [file 16-1894-Techapp-s1.pdf]

Article DOI: https://dx.doi.org/10.3201/eid2305.161894

Phenotypic and Genotypic Shifts in Hepatitis B Virus in Treatment-Naive Patients, Taiwan, 2008–2012

Technical Appendix

Technical Appendix Table 1. Demographic, clinical, and virologic characteristics of treatment-naive patients with chronic hepatitis B who received pretreatment assessments, Taiwan, 2008–2012*

							Univariate		Multivariate	
Characteristic	All, n = 1,224	2008, n = 120	2009, n = 217	2010, n = 281	2011, n = 289	2012, n = 317	Coefficient	p value	Adjusted coefficient	p value
Age, y	48.98 ± 12.74	47.36 ± 11.87	46.98 ± 12.75	48.67 ± 12.90	49.86 ± 12.44	50.43 ± 12.98	0.010	**0.001**	0.007	**0.025**
Male	859 (70.18%)	97 (80.83%)	156 (71.89%)	183 (65.12%)	200 (69.20%)	223 (70.35%)	−0.121	0.137		
Cirrhosis present	243 (22.46%)	20 (20.83%)	39 (20.42%)	53 (21.37%)	63 (24.71%)	68 (23.29%)	0.093	0.322		
Platelet count, × 10^9/L	174.68 ± 69.89	182.55 ± 65.24	180.31 ± 58.22	174.78 ± 66.29	169.86 ± 76.72	172.54 ± 75.07	−0.001	0.132		
HBeAg status										
HBeAg-Positive	398 (32.52%)	40 (33.33%)	90 (41.47%)	107 (38.08%)	75 (25.95%)	86 (27.13%)	−0.277	**<0.001**	−0.199	**0.021**
Anti-HBe-Positive	945 (77.21%)	93 (77.50%)	147 (67.74%)	212 (75.44%)	238 (82.35%)	255 (80.44%)	0.256	0.004†		
HBV Genotype										
B	940 (76.80%)	89 (74.17%)	159 (73.27%)	213 (76.07%)	232 (80.28%)	247 (77.92%)	0.142	0.109		
C	265 (21.65%)	27 (22.50%)	45 (20.74%)	66 (23.57%)	57 (19.72%)	70 (22.08%)	−0.014	0.874		
HBV BCP mutation										
1719	78 (6.37%)	8 (6.67%)	13 (5.99%)	22 (7.83%)	20 (6.92%)	15 (4.73%)	−0.119	0.435		
1728	12 (0.98%)	2 (1.67%)	4 (1.84%)	4 (1.42%)	0 (0.00%)	2 (0.63%)	−0.721	0.056		
1730	295 (24.10%)	33 (27.50%)	63 (29.03%)	69 (24.56%)	60 (20.76%)	70 (22.08%)	−0.185	**0.034‡**		
1752	432 (35.29%)	47 (39.17%)	76 (35.02%)	110 (39.15%)	87 (30.10%)	112 (35.33%)	0.084	0.282		
1753	163 (13.32%)	12 (10.00%)	30 (13.82%)	39 (13.88%)	44 (15.22%)	38 (11.99%)	0.028	0.799		
1762	536 (43.79%)	53 (44.17%)	89 (41.01%)	122 (43.42%)	131 (45.33%)	141 (44.48%)	0.046	0.538		
1764	561 (45.83%)	56 (46.67%)	93 (42.86%)	132 (46.98%)	133 (46.02%)	147 (46.37%)	0.028	0.711		
1766	81 (6.05%)	9 (8.33%)	9 (6.45%)	23 (7.12%)	15 (5.19%)	25 (4.73%)	0.095	0.528		
1768	74 (6.05%)	10 (8.33%)	14 (6.45%)	20 (7.12%)	15 (5.19%)	15 (4.73%)	−0.247	0.114		
1799	942 (76.96%)	89 (74.17%)	160 (73.73%)	212 (75.44%)	233 (80.62%)	248 (78.23%)	0.149	0.092		
HBV precore stop-codon mutations										
1896	854 (69.77%)	81 (67.50%)	139 (64.06%)	181 (64.41%)	217 (75.09%)	236 (74.45%)	0.244	0.003*		
1899	290 (23.69%)	24 (20.00%)	41 (18.89%)	65 (23.13%)	75 (25.95%)	85 (26.81%)	0.206	0.019*		
HBV DNA, log copies/mL	7.14 ± 1.59	7.01 ± 1.60	7.14 ± 1.62	7.42 ± 1.56	7.09 ± 1.56	6.99 ± 1.61	−0.026	0.274		

*Bold font, p<0.05. BCP, basal core promoter; HBV, hepatitis B virus.
†Not included for multivariate analysis because anti-HBe and precore stop-codon mutations were tightly associated with HBeAg status.
‡Multivariate analysis including G1730C, HBeAg and Age resulted in adjusted p = 0.222, 0.049 and 0.027 respectively.

Technical Appendix Table 2. Clinical and virologic characteristics of 398 treatment-naive, HBeAg-positive patients who received pretreatment assessments, Taiwan, 2008–2012*

							Univariate		Multivariate	
Characteristic	All, n = 398	2008, n = 40	2009, n = 90	2010, n = 107	2011, n = 75	2012, n = 86	Coefficient	p value	Adjusted coefficient	p value
Age, y	41.69 ± 12.23	39.90 ± 11.10	41.63 ± 13.68	41.39 ± 11.41	42.05 ± 10.94	42.63 ± 13.33	0.006	0.281		
Male	257 (64.57%)	31 (77.50%)	55 (61.11%)	66 (61.68%)	47 (62.67%)	58 (67.44%)	–0.041	0.762		
Cirrhosis present	60 (%)	7 (20.00%)	10 (11.90%)	19 (20.43%)	10 (15.15%)	14 (16.87%)	0.021	0.91		
Platelet count, × 10^9/L	183.47 ± 68.23	193.12 ± 81.35	201.43 ± 53.65	172.81 ± 68.53	176.41 ± 70.22	180.71 ± 71.22	–0.002	0.118		
HBV genotype										
B	242 (60.80%)	21 (52.50%)	59 (65.56%)	68 (64.15%)	47 (62.67%)	47 (54.65%)	–0.073	0.579		
C	150 (37.69%)	18 (45.00%)	28 (31.33%)	37 (34.91%)	28 (37.33%)	39 (45.35%)	0.138	0.299		
HBV BCP mutations										
1719	35 (8.79%)	3 (7.50%)	9 (10.00%)	11 (10.28%)	5 (6.67%)	7 (8.14%)	–0.087	0.703		
1728	5 (1.26%)	0 (0.00%)	2 (2.22%)	3 (2.80%)	0 (0.00%)	0 (0.00%)	–0.601	0.299		
1730	160 (40.20%)	19 (47.50%)	35 (38.89%)	40 (37.38%)	28 (37.33%)	38 (44.19%)	0.000	0.997		
1752	137 (34.42%)	11 (27.50%)	36 (40.00%)	40 (37.38%)	18 (24.00%)	32 (37.21%)	–0.028	0.837		
1753	49 (12.31%)	3 (7.50%)	10 (11.11%)	10 (9.35%)	13 (17.33%)	13 (15.12%)	0.315	0.108		
1762	182 (45.73%)	18 (45.00%)	36 (40.00%)	47 (43.93%)	38 (50.67%)	43 (50.00%)	0.170	0.189		
1764	194 (48.74%)	19 (47.50%)	38 (42.22%)	53 (49.53%)	39 (52.00%)	45 (52.33%)	0.156	0.227		
1766	31 (7.79%)	4 (10.00%)	6 (6.67%)	13 (12.15%)	5 (6.67%)	3 (3.49%)	–0.315	0.19		
1768	23 (5.78%)	4 (10.00%)	3 (3.33%)	8 (7.48%)	6 (8.00%)	2 (2.33%)	–0.251	0.362		
1799	243 (61.06%)	21 (52.50%)	60 (66.67%)	68 (63.55%)	46 (61.33%)	48 (55.81%)	0.074	0.575		
HBV precore stop-codon mutations										
1896	170 (42.71%)	15 (37.50%)	37 (41.11%)	42 (39.25%)	35 (46.67%)	41 (47.67%)	0.176	0.177		
1899	41 (10.30%)	1 (2.50%)	9 (10.00%)	11 (10.28%)	5 (6.67%)	15 (17.44%)	0.437	**0.039**	0.558	**0.009**
HBV DNA (log copies/ml)	8.18 ± 1.27	7.74 ± 1.36	8.00 ± 1.41	8.32 ± 1.03	8.31 ± 1.26	8.30 ± 1.31	0.127	**0.013**	0.153	**0.003**

*Bold font, $p < 0.05$; BCP, basal core promoter; HBV, hepatitis B virus.
